# Acute Pancreatitis Management and Scoring in the Department of General Surgery, Northwick Park Hospital, 2022-2024

**DOI:** 10.7759/cureus.100172

**Published:** 2025-12-27

**Authors:** Bakhtawar Awan, Mohamed Elsaigh, Josef Watfah

**Affiliations:** 1 General Surgery, Northwick Park Hospital, London, GBR; 2 General and Emergency Surgery, Northwick Park Hospital, London, GBR

**Keywords:** atlanta, glasgow, marshall university, pancreatitis, quality improvement

## Abstract

Background and aim: Acute pancreatitis is a common cause of acute abdominal admissions, which usually resolve on their own; however, early severity stratification guides escalation of care and resource utilization. Early and accurate severity assessment using standardized scoring systems, such as the modified Atlanta classification, is crucial for effective clinical management and improved outcomes in patients with pancreatitis. Therefore, we conducted this clinical audit to highlight the use of the Atlanta scoring system. We designed this closed-loop audit to bridge the gap between national recommendations and clinical practice, with a focus on improving early risk stratification and patient management for acute pancreatitis at Northwick Park Hospital.

Methods: This study was a retrospective analysis conducted at Northwick Park Hospital, Department of General Surgery. Our audit was done over two separate cycles. The first cycle included 64 patients in 2023 from Epro and Cerner, while the second cycle included 80 patients in 2024 from Cerner. Between the two cycles, several interventions were introduced, including departmental teaching, visual prompts such as posters, and digital communications via email and messaging platforms. Scoring practices included the modified Atlanta score, the Modified Marshall scoring system, and the Glasgow-Imrie score. The audit was conducted in accordance with National Institute for Health and Care Excellence (NICE) NG104 guidelines and was approved through local clinical governance meetings for both audit cycles, with the consultant's agreement to implement updated scoring protocols.

Results: The first audit cycle in 2023 revealed that only 30% of patients had documented scoring on admission, and none had repeated scoring at 48 hours. The second cycle in 2024 demonstrated improvement, with admission scores increasing from 30% to 40%. Atlanta classification scoring on admission and 48 hours post-admission increased from 0% in the first cycle to 7.5% in the second cycle.

Conclusion: This quality improvement initiative demonstrated that targeted interventions significantly enhance adherence to national scoring protocols, thereby improving standardized acute pancreatitis care. However, continuous attention must be paid to assess and improve the use of severity scoring systems in the management of acute pancreatitis, in addition to increasing the frequency and consistency of applying the Revised Atlanta Classification at the time of admission and 48 hours post-admission.

## Introduction

Among gastrointestinal illnesses, acute pancreatitis is the most prevalent cause of hospitalization globally. It is related to acute pancreatic inflammation [1]. Acute pancreatitis can range greatly in severity, from mild cases that require conservative care to more complex, severe situations that have a high morbidity and fatality rate. Acute pancreatitis is becoming more common [2,3]. The rise is partially attributable to elevated metabolic syndrome, hypertriglyceridemia, and alcohol consumption, regardless of whether this trend is associated with a real increase in incidence or increased detection [4]. Acute pancreatitis affects approximately 56 individuals per 100000 annually in the UK, while chronic pancreatitis has an estimated incidence of five per 100000 in Western Europe, although this likely represents an underestimate [5].

The UK BioBank cohort demonstrated a significant disease burden, with a prevalence of chronic pancreatitis reaching 163 per 100000 population, while the incidence of acute pancreatitis showed a marked increase from 21.4 per 100000 annually during 2001-2005 to 48.2 per 100000 between 2016 and 2020 [6].

Acute presentation is easy to diagnose, but predicting the course and outcome of the disease is a significant challenge. When determining the appropriate treatment duration, the length of the illness is crucial [7-9]. Patients typically present with moderate to severe epigastric pain accompanied by nausea and anorexia, with pain characteristics varying according to underlying etiology. Biliary causes characteristically produce sharp, sudden-onset pain radiating to the back, while metabolic and toxicologic etiologies tend to manifest with more gradual, diffuse epigastric discomfort [10]. In contrast, metabolic and toxicologic etiologies, particularly alcohol-induced pancreatitis, typically develop more insidiously with dull, diffuse epigastric pain that lacks the characteristic radiation pattern. Obtaining a detailed family history is important, especially when more common etiologies appear less likely, as there are rare genetic cases of familial pancreatitis [11].

In cases of fever, tachycardia, and more severe conditions, such as hypotension, a physical examination is frequently crucial for assessing severity [12]. Typically, the abdominal exam reveals rigidity, decreased bowel sounds, and epigastric discomfort, often accompanied by potential guarding. When there has been severe retroperitoneal bleeding, the Grey-Turner's sign may manifest as flank ecchymosis. On the other hand, Cullen's sign manifests as peritoneal hemorrhage-related periumbilical ecchymosis [13]. The Revised Atlanta Classification states that in order to diagnose acute pancreatitis, at least two of the three requirements must be met: abdominal pain that is consistent with pancreatitis, abdominal imaging that is compatible with acute pancreatitis, and a lipase or amylase level that is three times the upper limit of normal [14].

Despite clear national guidance, preliminary observations at our institution suggested suboptimal compliance with severity scoring protocols, highlighting a gap between evidence-based recommendations and routine clinical practice. The aim of our audit was to achieve optimal management of patients with acute pancreatitis at Northwick Park Hospital through the systematic implementation of evidence-based severity assessment protocols. We also set our objective to improve compliance with UK national guidelines by enhancing the utilization of the Atlanta scoring system for severity classification of acute pancreatitis, thereby standardizing clinical practice and improving early risk stratification.

## Materials and methods

Standards

Our study was a retrospective analysis conducted at the Department of General Surgery, Northwick Park Hospital. This retrospective audit was conducted in full compliance with National Institute for Health and Care Excellence (NICE) Guideline NG104 on Pancreatitis (2018), which provides evidence-based recommendations for managing acute and chronic pancreatitis [15]. This retrospective clinical audit was formally registered and approved by the Northwick Park Hospital Clinical Governance Committee under audit registration number SUR.NP.24.333. As a retrospective clinical audit, this work was exempt from Research Ethics Committee (IRB) review. Individual patient informed consent was waived, as the audit involved only the anonymized evaluation of existing medical records with no patient interventions, in compliance with the London North West University Healthcare NHS Trust Privacy Notice and the NHS England Confidentiality Policy.

Data collection

Given the retrospective nature of this audit, a post-hoc power calculation was performed. With sample sizes of 64 (cycle 1) and 80 (cycle 2) patients achieved, and an observed improvement from 30% to 40% in overall scoring compliance, our study demonstrated 72% power to detect this difference at α=0.05. While this is below the conventional 80% threshold, it represents a reasonable power for a quality improvement initiative in a real-world clinical setting.

**Figure 1:**
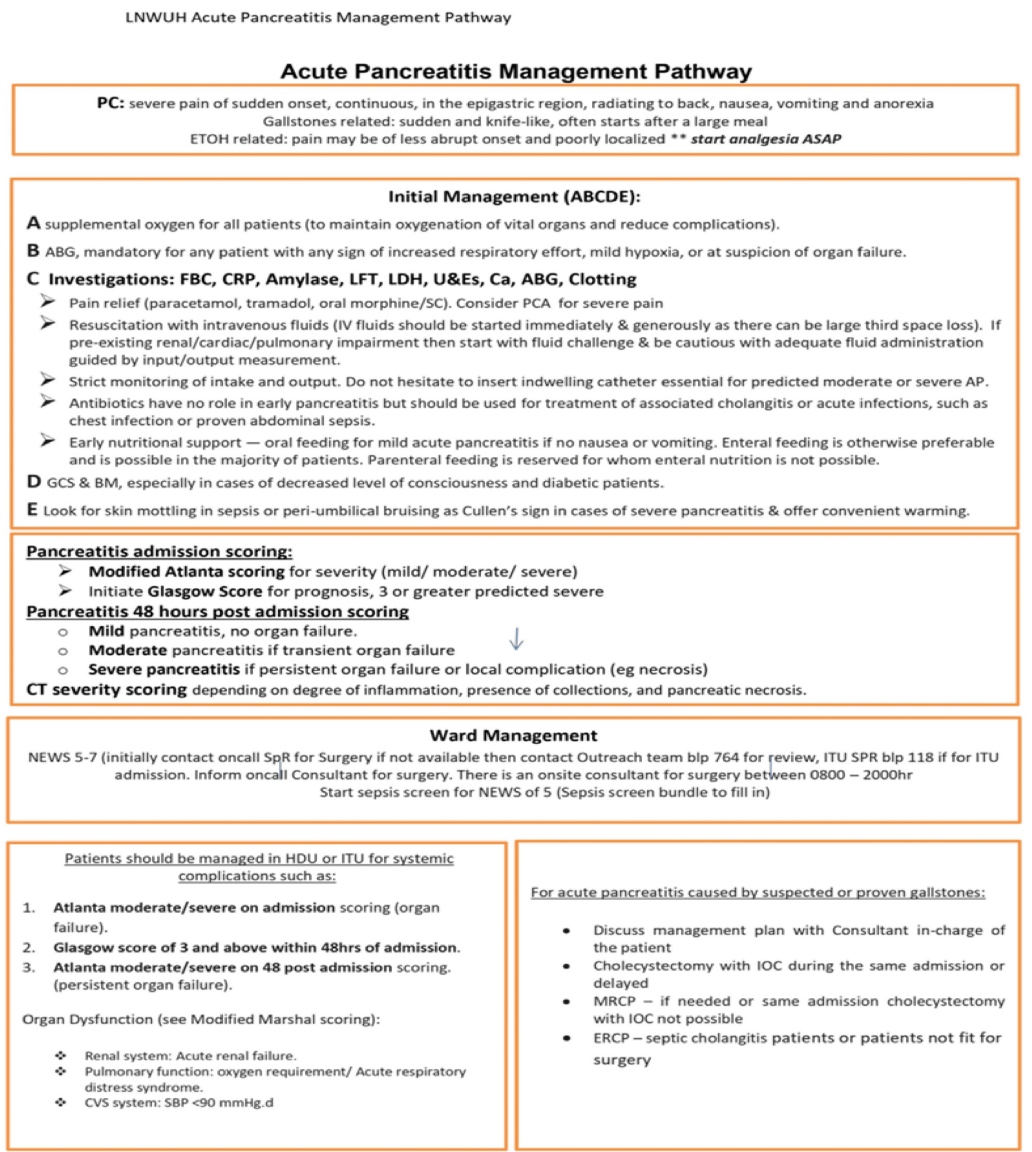
Acute pancreatitis management pathway Clinical management protocol for acute pancreatitis implemented at Northwick Park Hospital in accordance with NICE guideline NG104. The pathway outlines: (1) presenting clinical features differentiating gallstone-related (sudden, knife-like pain) versus alcohol-related (gradual onset) presentations; (2) initial ABCDE management, including oxygen supplementation, comprehensive investigations (FBC, CRP, amylase, LFTs, LDH, U&Es, calcium, ABG,  clotting), pain management, IV fluid resuscitation, and early nutritional support; (3) severity scoring systems, including the Modified Atlanta Classification on admission (mild/moderate/severe), the Glasgow–Imrie score for prognosis (≥3 predicts severe disease), and mandatory re-scoring at 48 hours to assess disease progression and guide management; (4) ward management protocols with NEWS-based escalation criteria (5-7 for surgical registrar review, ≥8 for ITU assessment) and HDU/ITU admission criteria based on Atlanta classification and organ dysfunction (Modified Marshall scoring). Gallstone-related cases require discussion for cholecystectomy planning, while ERCP is reserved for septic cholangitis or surgical contraindications. NICE: National Institute for Health and Care Excellence; FBC: full blood count; CRP: C-reactive protein; LFTs: liver function tests; LDH: lactate dehydrogenase; U&Es: urea and electrolytes; ABG: arterial blood gas; NEWS: National early warning score; ITU: intensive therapy unit; HDU: high dependency unit; GCS: Glasgow coma scale; BM: blood glucose monitoring; PCA: patient-controlled analgesia; ERCP: endoscopic retrograde cholangiopancreatography; SPR: surgical registrar; IOC: intraoperative cholangiogram; MRCP: magnetic resonance cholangiopancreatography; LNWUH: London North West University Healthcare

**Figure 2:**
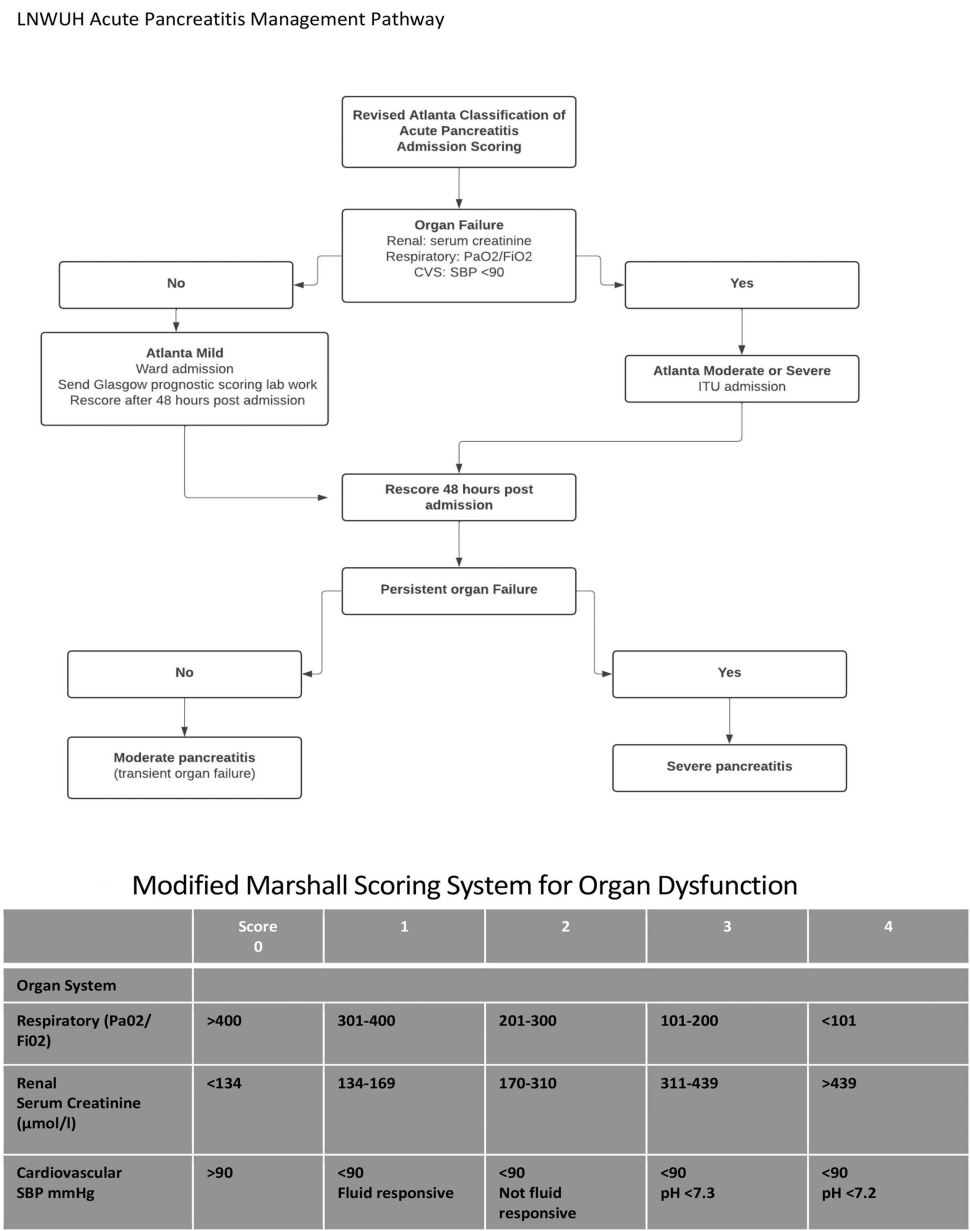
Modified Marshall scoring system Decision algorithm for acute pancreatitis severity classification using the Revised Atlanta Classification. Patients are assessed for organ failure based on three criteria: renal function (serum creatinine), respiratory function (PaO₂/FiO₂ ratio), and cardiovascular status (systolic blood pressure). Absence of organ failure leads to Atlanta mild classification with ward admission and Glasgow scoring, while the presence of organ failure requires ITU admission for Atlanta moderate or severe classification. Re-scoring at 48 hours post-admission differentiates moderate pancreatitis (transient organ failure <48 hours) from severe pancreatitis (persistent organ failure ≥48 hours). The Modified Marshall scoring table grades organ dysfunction on a 0-4 scale across respiratory (PaO₂/FiO₂: >400 to <101), renal (creatinine: <134 to >439 μmol/L), and cardiovascular systems (SBP: >90 to <90 mmHg with pH considerations). A score ≥2 in any system defines organ failure and guides clinical management decisions. ITU: intensive therapy unit; SBP: systolic blood pressure; PaO₂: arterial partial pressure of oxygen; FiO₂: fraction of inspired oxygen; LNWUH: London North West University Healthcare

Our audit was done over two separate cycles. The first cycle included 64 patients in 2023 from Epro and Cerner, while the second cycle included 80 patients in 2024 from Cerner. Between the two cycles, several interventions were introduced, including departmental teaching and digital communications via email and messaging platforms. Importantly, the trust guidelines were also updated to emphasize the recommended scoring practices either at admission or 48 hours post-admission. Scoring practices included the modified Atlanta score [16], the Modified Marshall scoring system [17], and the Glasgow-Imrie score (Figures 1, 2) [18].

Audit criteria

Data was retrospectively collected on patients admitted and managed for acute pancreatitis at Northwick Park Hospital in the Department of General Surgery. No exclusion criteria were reported.

Data analysis

Data were collected and registered for further statistical analysis. Analysis was done using the Statistical Package for Social Sciences (SPSS) software program (version 26) (IBM Corp., Armonk, NY). Qualitative variables were recorded as frequencies and percentages.

## Results

**Figure 3:**
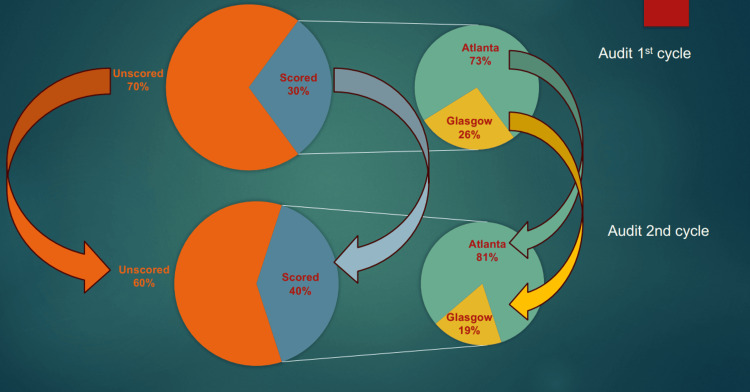
Findings of first and second cycles of our audit

**Figure 4:**
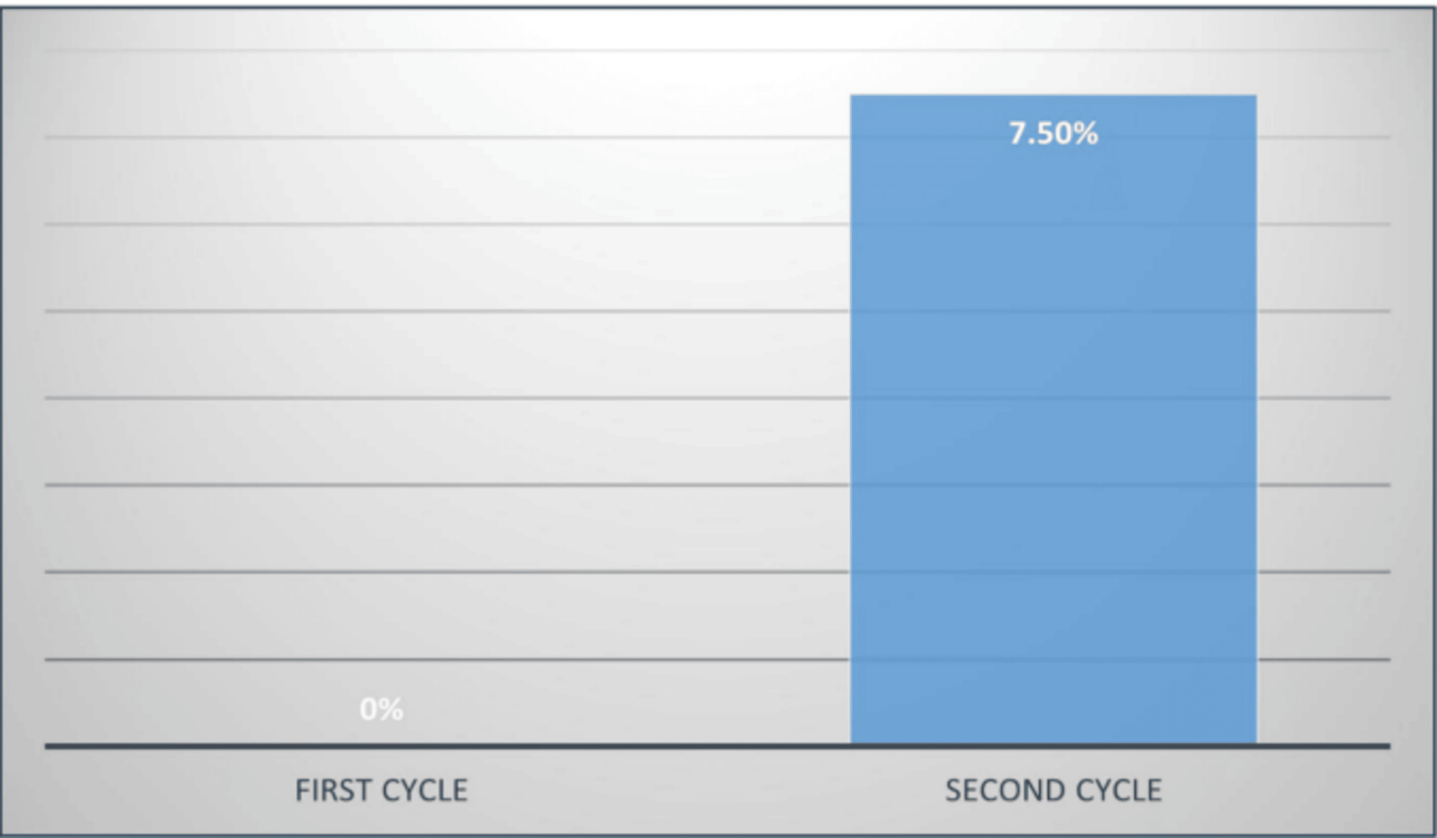
Atlanta classification scoring on admission and 48 hours post-admission In the first and second cycles of our audit. It went up from 0% in the first cycle to 7.5% in the second cycle.

The first audit cycle in 2023, involving 64 patients, showed that only 30% had documented scoring on admission, and none had repeated scoring at 48 hours. The second cycle, in 2024, involving 80 patients, demonstrated improvement, with scoring on admission increasing by 10% (Figure 3). Atlanta classification scoring on admission and 48 hours post-admission increased from 0% in the first cycle to 7.5% in the second cycle (Figure 4).

## Discussion

Our audit demonstrated modest but measurable improvement in scoring compliance, with overall documentation increasing from 30% to 40% between cycles. Most significantly, Atlanta classification utilization improved from 0% to 7.5%, representing the successful introduction of a previously absent practice. However, these results indicate substantial room for improvement, as 60% of patients still lack documented severity scoring. The modest improvement to 40% compliance, despite comprehensive interventions including strategic poster placement across the surgical assessment unit, accident and emergency (A&E), and intensive therapy unit (ITU), as well as dedicated teaching sessions, demonstrates that guidelines are not being strictly followed in practice. With 60% of patients still lacking documented scoring in cycle 2, significant implementation barriers persist. This suboptimal adherence highlights the need for more stringent measures, including mandatory documentation requirements and enhanced accountability systems, to achieve the strict guideline compliance necessary for optimal patient care. Although acute pancreatitis is a frequent abdominal illness that usually resolves on its own, some cases have a poor outcome that requires surgery to treat the systemic and local consequences [8]. Our audit represents a closed-loop quality improvement project aimed at aligning clinical practice with national standards and improving the early identification and management of patients with acute pancreatitis. The modified Atlanta classification is a precise, affordable, and practical method of categorizing acute pancreatitis by dividing it into mild, moderate, and severe groups while taking clinical, laboratory, and radiographic factors into account [14].

Only two forms of acute pancreatitis were identified by the initial classification system, which mostly relied on the presence or absence of organ failure to determine whether the condition was mild or severe [9]. In order to compile the missing phase of acute pancreatitis that has been found to be producing substantial morbidity based on emerging systemic problems, this additionally specifies a third category of moderate pancreatitis [6]. According to observations, mild pancreatitis does not exhibit these alterations in terms of the incidence of organ failure. As the illness worsens, these alterations appear in the intermediate and severe stages of pancreatitis [7]. Additionally, the recently created category of "moderately severe acute pancreatitis" is thought to result in problems that are either local or systemic in character, as well as temporary organ failure with a timeline of less than two days or 48 hours. The local ones consist of fluid accumulations within or near the pancreas. However, the systemic ones are those that, when they progress, result in comorbid conditions [11,15]. These are known to happen in the second week of acute pancreatitis in general, and they are clinically suspected when a patient exhibits recurrent abdominal pain along with elevated pancreatic enzyme levels, which may or may not result in sepsis and/or organ failure at the same time [19].

If organ failure persists for more than 48 hours or two days, it may be assumed that acute pancreatitis has worsened. To address this issue, the Revised Atlanta Classification now incorporates the Modified Marshall scoring system for assessing organ failure [20]. The measurement of the partial pressure of oxygen/fraction of inspired oxygen, which is expressed as ratio of arterial oxygen partial pressure to fractional inspired oxygen (PaO_2_/FiO_2)_, serum creatinine levels in relation to the renal excretory system's condition, and the systolic blood pressure in millimeters-Hg are all taken into consideration [21].

This study has several important limitations. First, the retrospective design limits our ability to establish causal relationships between interventions and observed improvements. Second, the absence of a control group prevents definitive attribution of changes to our specific interventions rather than external factors. Third, our sample sizes may have been insufficient to detect smaller but clinically meaningful improvements in scoring compliance. Fourth, the single-center design limits generalizability to other healthcare settings. Finally, our audit focused on process measures (scoring documentation) rather than patient outcomes, limiting assessment of clinical impact. Study strengths include a design that enables systematic improvement, formal clinical governance approval, adherence to NICE guidelines, multi-modal interventions addressing implementation barriers, and a focus on nationally validated scoring systems.

## Conclusions

While the diagnosis of acute pancreatitis is straightforward, our audit confirms that the major clinical challenge lies in accurately predicting disease progression and determining appropriate levels of care. Our findings demonstrate that despite targeted interventions, significant gaps persist in implementing standardized scoring protocols, with only 40% of patients at admission receiving documented severity assessment. We also conclude that continuous attention must be paid to assess and improve the use of severity scoring systems in the management of acute pancreatitis, thereby promoting adherence to UK national guidelines. Particular emphasis must be placed on increasing both the frequency and consistency of applying the Revised Atlanta Classification at admission and 48 hours post-admission, as these critical time points enable early identification of patients at risk for complications and facilitate timely interventions.

## References

[1] Beij A, Verdonk RC, van Santvoort HC, de-Madaria E, Voermans RP (2025). Acute pancreatitis: an update of evidence-based management and recent trends in treatment strategies. United European Gastroenterol J.

[2] Szatmary P, Grammatikopoulos T, Cai W (2022). Acute pancreatitis: diagnosis and treatment. Drugs.

[3] Mederos MA, Reber HA, Girgis MD (2021). Acute pancreatitis: a review. J Am Med Assoc.

[4] Iannuzzi JP, King JA, Leong JH (2022). Global incidence of acute pancreatitis is increasing over time: a systematic review and meta-analysis. Gastroenterology.

[5] (2020). Pancreatitis. https://www.nice.org.uk/guidance/ng104/chapter/Context.

[6] Spagnolo DM, Greer PJ, Ohlsen CS (2022). Acute and chronic pancreatitis disease prevalence, classification, and comorbidities: a cohort study of the UK Biobank. Clin Transl Gastroenterol.

[7] Walkowska J, Zielinska N, Tubbs RS, Podgórski M, Dłubek-Ruxer J, Olewnik Ł (2022). Diagnosis and treatment of acute pancreatitis. Diagnostics (Basel).

[8] Zerem E, Kurtcehajic A, Kunosić S, Zerem Malkočević D, Zerem O (2023). Current trends in acute pancreatitis: diagnostic and therapeutic challenges. World J Gastroenterol.

[9] Jaber S, Garnier M, Asehnoune K (2022). Guidelines for the management of patients with severe acute pancreatitis, 2021. Anaesth Crit Care Pain Med.

[10] Van den Berg FF, Boermeester MA (2023). Update on the management of acute pancreatitis. Curr Opin Crit Care.

[11] Zheng Z, Ding YX, Qu YX, Cao F, Li F (2021). A narrative review of acute pancreatitis and its diagnosis, pathogenetic mechanism, and management. Ann Transl Med.

[12] Li CL, Jiang M, Pan CQ, Li J, Xu LG (2021). The global, regional, and national burden of acute pancreatitis in 204 countries and territories, 1990-2019. BMC Gastroenterol.

[13] Heckler M, Hackert T, Hu K, Halloran CM, Büchler MW, Neoptolemos JP (2021). Severe acute pancreatitis: surgical indications and treatment. Langenbecks Arch Surg.

[14] Alves JR, Ferrazza GH, Nunes Junior IN, Teive MB (2021). The acceptance of changes in the management of patients with acute pancreatitis after the revised Atlanta classification. Arq Gastroenterol.

[15] Samarasekera E, Mahammed S, Carlisle S, Charnley R (2018). Pancreatitis: summary of NICE guidance. Br Med J.

[16] Banks PA, Bollen TL, Dervenis C (2013). Classification of acute pancreatitis-2012: revision of the Atlanta classification and definitions by international consensus. Gut.

[17] Marshall JC, Cook DJ, Christou NV, Bernard GR, Sprung CL, Sibbald WJ (1995). Multiple organ dysfunction score: a reliable descriptor of a complex clinical outcome. Crit Care Med.

[18] Blamey SL, Imrie CW, O'Neill J, Gilmour WH, Carter DC (1984). Prognostic factors in acute pancreatitis. Gut.

[19] Singh V, Dhande RP, Mishra G (2021). Atlanta classification for acute pancreatitis-a study protocol. J Pharm Res Int.

[20] Marshall JC (1994). A scoring system for multiple organ dysfunction syndrome. Sepsis: Update in Intensive Care and Emergency Medicine.

[21] Maringhini A, Rossi M, Patti R, Maringhini M, Vassallo V (2024). Acute pancreatitis during and after pregnancy: a review. J Clin Med.

